# LPCAT1 as a prognostic biomarker and risk indicator for hepatocellular carcinoma: insights into genes related to lipid metabolism

**DOI:** 10.3389/fonc.2026.1707270

**Published:** 2026-01-30

**Authors:** Hang Zhai, Jicai Wang, Yongfei He, Shengjie Hong, Kai Huang, Shuai Hu, Junming Xu, Shuang Hao, Guangquan Zhang, Xianjie Shi

**Affiliations:** 1 Department of Hepatobiliary and Pancreatic Surgery, The Eighth Affiliated Hospital, Sun Yat-sen University, Shenzhen, China; 2 Department of Hepatobiliary Surgery, The First Affiliated Hospital of Guangxi Medical University, Nanning, Guangxi, China

**Keywords:** hepatocellular carcinoma, lipid metabolism, LPCAT1, prognostic biomarker, targeted therapy and immunotherapy

## Abstract

**Background:**

Hepatocellular carcinoma (HCC) is a lethal malignancy. Lipid metabolic reprogramming influences therapy response, but its prognostic value is undefined.

**Methods:**

From TCGA and GEO data, we derived an 8-gene lipid metabolism-related risk score (LMrisk) via LASSO regression. Its associations with tumor immunity and drug sensitivity were analyzed. The lead gene, *LPCAT1*, was functionally assessed *in vitro*.

**Results:**

The LMrisk signature robustly stratified patient survival in both cohorts. Integrated analysis identified *LPCAT1* as a hub gene. Its overexpression in HCC cells potently enhanced proliferation, migration, and invasion.

**Conclusion:**

*LPCAT1* is a vital prognostic biomarker and putative therapeutic target in HCC.

## Introduction

Hepatocellular carcinoma (HCC) is among the top three most common cancers globally, and its incidence and mortality rates are on the rise (1, 2). HCC exhibits histopathological diversity and various histological subtypes, highlighting its heterogeneity (3). This heterogeneity arises from changes in multiple signaling pathways (3, 4). Understanding its temporal classification, molecular features, and clinical significance remains an active area of research. Among tumor characteristics, abnormal molecular features are a primary target for HCC treatment strategies (3–8).

Lipid metabolic reprogramming drives tumor proliferation, angiogenesis, and metastasis (9–16). Metabolic reprogramming in tumor cells elicits metabolic strain in immune and stromal cells infiltrating the tumor, impairing antitumoral immune responses (17, 18). Lysophosphatidylcholine acyltransferase 1 (*LPCAT1*) belongs to the phosphatidylcholine acyltransferase (PCAT) family and plays an integral role in lipid metabolism by facilitating the modification of cell membrane phosphatidylcholine (PC) (19–21). After *de novo* synthesis, PC molecules have their sn-2 fatty acyl chains hydrolyzed to produce lysophosphatidylcholine (LPC), which are subsequently reacylated by LPCATs to reintegrate fatty acids at the sn-2 position, generating new PC species (19, 20). Moreover, LPCAT1 predominantly utilizes 16:0-CoA (palmitoyl-CoA) as its substrate in catalyzing the creation of dipalmitoyl phosphatidylcholine (22). Dipalmitoyl phosphatidylcholine facilitates signal transduction from the membrane to the cytosol by acting as a key intermediate in cellular signaling pathways (22). However, its specific role in driving the immunosuppressive HCC microenvironment and its value as a therapeutic target remain unclear.

In this study, we developed a lipid metabolism-related prognostic risk score specific to HCC, with the TCGA cohort as the training set, and confirmed its robust prognostic significance in the GSE14520 validation cohort. The LMrisk score shows a significant correlation with signatures of immune cell infiltration and expression of immune checkpoints, functioning as a new biomarker predictive of immunotherapy response. Elevated LMrisk scores indicate an immunosuppressive microenvironment and treatment refractoriness. Our findings may help confirm the link between elevated *LPCAT1* expression and HCC progression, providing new targets for improving liver cancer immunotherapy.

## Materials and methods

### Data and resources

RNA-seq data with matched clinical annotations from HCC patients were acquired from The Cancer Genome Atlas (TCGA, https://portal.gdc.cancer.gov). 369 HCC and 50 normal liver samples underwent analysis within the TCGA dataset. RNA expression profiles and clinical details from the GSE14520 dataset were retrieved via the Affymetrix Human Genome U133A 2.0 Array platform from the GEO database (https://www.ncbi.nlm.nih.gov/geo/). LMGs were curated and sourced from Reactome (https://reactome.org/download-data/). Single-cell RNA sequencing (scRNA-seq) data for core LMGs in hepatocellular carcinoma (HCC) were analyzed via the online tool accessible at: http://zhangninglab.com:3838/scPLC/. Additionally, spatial transcriptome analysis for each key LMG was performed using the spatial transcriptomic data from HCCDB v2.0 available at http://lifeome.net:809/.

### Analysis of differential expression in LMGs and functional enrichment

The ‘edgeR’ package identified differentially expressed LMGs in tumor versus normal tissues, with criteria of an adjusted P-value < 0.05 and a fold change > 1.0. The protein-protein interaction network of these differentially expressed LMGs was constructed via the STRING database (https://string-db.org/). The clusterProfiler package conducted GO and KEGG (23–25) enrichment analyses for differentially expressed LMGs, with statistical significance defined as a false discovery rate < 0.05. The complete results of the GO and KEGG enrichment analyses, including FDR-adjusted p-values, are available in **Supplementary Table S3**.

### Development and verification of the lipid metabolism-related risk score

Univariate Cox regression identified statistically significant prognosis-related LMGs (P < 0.05). LASSO regression via the “glmnet” R package was applied to these prognosis-linked LMGs to develop a prognostic model for HCC patients. The lipid metabolism-related risk score (LMrisk) was computed by multiplying normalized expression levels of selected signature genes by their respective regression coefficients, formulated as: LMrisk = ∑ (gene expression × gene coefficient). High/low-risk stratification of TCGA-HCC patients employed median LMrisk. Subsequent Kaplan-Meier analysis revealed survival differences. Multi-year (1/3/5) AUC values from “survivalROC” determined model accuracy. This model was further validated in the GSE14520 testing cohort. Through Cox regression modeling, LMrisk demonstrated independent prediction of HCC overall survival.

### Development and validation of the prognostic nomogram

A prognostic nomogram generated via the rms package estimated multi-year (1/3/5) OS probabilities. The survival package enabled time-dependent ROC analysis for predictive performance quantification. Its precision was checked using calibration curves from the ‘rms’ package. Survival outcome predictability of the nomogram was tested by time-dependent ROC.

### Analysis of immunological characteristics

CIBERSORT and xCell were employed to analyze immune cell composition and evaluate immune infiltration in hepatocellular carcinoma patients from the TCGA cohort. Heatmaps illustrate the distribution of immune cell infiltration levels across the LMrisk cohorts. Following this, we carried out a Spearman correlation analysis to examine the connections between lysophosphatidylcholine acyltransferase 1 (*LPCAT1*), Human serum paraoxonase-1 (*PON1*), Farnesyl diphosphate synthase (*FDPS*), Cathepsin A(*CTSA*), StAR-related lipid transfer domain containing 5 (*STARD5*), Cytochrome P450 2C9(*CYP2C9*), Cytochrome P450 2C19 (*CYP2C19*), and Thioredoxin reductase 1 (*TXNRD1*) and immune cell proportions, as well as microenvironment, immune, and stroma scores. To elucidate potential immunomodulatory mechanisms in HCC, the expression patterns of immune checkpoint genes were analyzed across distinct LMrisk subgroups. For analyses involving multiple comparisons—including the evaluation of differential immune cell infiltration and correlations between gene expression and immune features—p-values were adjusted for multiple testing using the Benjamini-Hochberg method to control the false discovery rate (FDR). Adjusted p-values (FDR) < 0.05 were considered statistically significant. Detailed statistical results for the analyses of immunological characteristics, including FDR-adjusted p-values, are compiled in **Supplementary Table S3**.

### Prediction of drug sensitivity

Based on GDSC (Genomics of Drug Sensitivity in Cancer), pRRophetic predicted drug sensitivity per cluster. The half-maximal inhibitory concentrations (IC50) of various chemotherapeutic drugs were determined to generate subgroup-specific drug response profiles, revealing differential sensitivity across clusters.

### Small molecule-protein docking

The three-dimensional structures of the small-molecule tyrosine kinase inhibitors (Sorafenib, Lenvatinib, and Regorafenib) were retrieved from the PubChem database. All ligand structures were prepared using Schrödinger’s Ligprep module under the OPLS_4 force field to generate possible ionization states, tautomers, and stereoisomers at physiological pH (7.0 ± 2.0) with the Epik module. The structure of human LPCAT1 (UniProt ID: Q8NF37), which lacks an experimental full-length structure, was obtained from the AlphaFold2 database and prepared using the Protein Preparation Wizard. This preparation included assigning bond orders, adding hydrogens, filling missing side chains, removing water molecules and cofactors, optimizing the hydrogen-bonding network, and performing energy minimization with the OPLS_4 force field. The binding site on LPCAT1 was predicted using the Sitemap tool. The top-ranked predicted site was selected for docking. To predict the binding modes of the small-molecule inhibitors, molecular docking was conducted using the Glide module in Standard Precision (SP) mode, a standard approach for predicting how small molecules bind to protein targets (26–28). The receptor grid was generated centered on the predicted binding site with an inner box size of 10 Å and an outer box size defined automatically to encompass the site. For each ligand, 10 poses were generated and subjected to post-docking minimization. The pose with the most favorable Glide docking score was selected as the optimal binding mode for subsequent analysis and visualization.

### Protein-protein docking and MM-GBSA calculation

The three-dimensional structures of the target proteins were prepared as follows: the full-length structure of human LPCAT1 (UniProt ID: Q8NF37) was obtained from the AlphaFold2 database; the crystal structures of human PD-1 (*PDCD1*, PDB: 6UMU), PD-L1 (*CD274*, PDB: 4Z18), and CTLA-4 (PDB: 3OSK) were retrieved from the Protein Data Bank. All structures were prepared using the Protein Preparation Wizard module in Schrödinger, which included assigning bond orders, adding hydrogen atoms, removing water molecules and cofactors, optimizing the hydrogen-bonding network, and performing energy minimization with the OPLS_4 force field. Protein-protein docking was performed using the Piper module, which is specifically designed for sampling macromolecular complexes (26, 29, 30). The standard protocol was employed with the number of rotational probes set to 70,000 to ensure thorough conformational sampling of the ligand protein. For each system, 10 docking poses were generated, with LPCAT1 defined as the receptor and PD-1, PD-L1, or CTLA-4 defined as the ligand. From the docking results, the top-ranked pose—selected from the largest cluster based on Piper’s clustering analysis—was chosen as the representative complex structure for each pair. Finally, to quantitatively estimate the binding affinity, the molecular mechanics/generalized Born surface area (MM-GBSA) method was applied to these representative complexes using the Prime module in Schrödinger. The binding free energy (ΔG) was calculated with the VSGB solvation model and OPLS_4 force field, and the final values are reported in kcal/mol.

### Molecular dynamics simulations

Molecular dynamics (MD) simulations were employed to investigate protein fluctuations and conformational changes. The root-mean-square deviation (RMSD) of Cα atoms relative to the initial structure was calculated over the simulation time to monitor system dynamics. To further evaluate binding mode stability, standard MD simulations were performed for three protein-ligand complexes and three protein-protein systems using the Desmond module (Schrödinger Suite 2023).

### Cell culture, clinical specimens, and transfection

The murine HCC cell line Hepa1–6 and all human HCC cell lines (MHCC97H, HepG2, Hep3B, Huh7, and MHCC97L) were obtained from the Cell Bank of the Chinese Academy of Sciences (Shanghai). Hepa1-6, MHCC97H, Huh7, and MHCC97L cells were cultured in Dulbecco’s Modified Eagle Medium (DMEM) supplemented with 10% fetal bovine serum (FBS) and 1% penicillin-streptomycin (P/S). HepG2 and Hep3B cells were cultured in Minimum Essential Medium (MEM) containing non-essential amino acids (NEAA), 10% FBS, and 1% P/S. All cells were maintained at 37°C in a humidified atmosphere with 5% CO_2_. Human HCC tissue specimens were procured from patients who provided written informed consent and were collected at a 1,500-bed tertiary care hospital. This study was carried out in accordance with the protocol approved by the Ethics Committee of The Eighth Affiliated Hospital of Sun Yat-sen University (Approval No. 2025-077-01). All procedures involving human participants were performed: (i) in accordance with the ethical standards of the institutional committee, (ii) The Declaration of Helsinki (1964) and its later amendments, (iii) Relevant national/international guidelines and regulations. Written informed consent was obtained from all individual participants included in the study. For gene knockdown, two independent small interfering RNAs (siRNAs) targeting *LPCAT1* (siLPCAT1–1 and siLPCAT1-2) were designed and synthesized by Tsingke (Beijing, China). The siRNA sequences were as follows: siLPCAT1-1, sense 5’-CCA GGA UUC UCG CAG GAA AdTdT-3’, antisense 5’-UUU CCU GCG AGA AUC CUG GdTdT-3’; siLPCAT1-2, sense 5’-GAA GAU AGG UAU UGC GGA GUU dTdT-3’, antisense 5’-AAC UCC GCA AUA CCU AUC UUC dTdT-3’. Transfection was performed using Lipofectamine 3000 reagent (Thermo Fisher Scientific, USA) according to the manufacturer’s instructions. A non-targeting siRNA (si-NC) was used as a negative control. For gene overexpression, lentiviral vectors encoding the *LPCAT1* gene and an empty control vector were obtained from RiboBio (Guangzhou, China). HCC cells were transduced with the lentiviral particles according to the manufacturer’s protocol, followed by selection with puromycin to establish stable cell lines. The sequences targeted for PCR or gene editing are detailed in **Supplementary Table S1** and **Supplementary Table S2**.

### Immunohistochemistry and western blotting

Paraffin-embedded HCC samples were collected from patients following curative hepatectomy. For paired analysis, 10 pairs of HCC tumors and matched adjacent non-tumor liver tissues were used. For stage-specific comparison, tumor tissues from 8 patients with TNM stage I and 9 patients with stage III were included. Tissue sections (4 μm thick) were stained using a monoclonal antibody against LPCAT1 (1:200, 66044-1-Ig, Proteintech). Quantification of staining intensity was performed by measuring the Average Optical Density (AOD) using ImageJ software. The integrated optical density (IOD) of the DAB-positive area and the total measured area were recorded. The AOD for each sample was calculated as AOD = IOD/Area. Western blotting was conducted according to standard protocols. Protein band intensities were quantified using ImageJ software, and the relative expression of LPCAT1 was normalized to β-actin. The antibodies employed were sourced from these suppliers: LPCAT1 (1:5000, 66044-1-Ig, Proteintech), β-actin (1:20000, AC038, Abclonal).

### Colony formation assay

HCC cells were plated at a density of 1,000 cells/well in 6-well plates with DMEM supplemented with 10% FBS, then incubated at 37°C in a 5% CO_2_ atmosphere. Following a three-week culture period, colonies were fixed and stained with 0.5% crystal violet for 30 minutes to evaluate colony-forming ability.

### EdU proliferation assay

HCC cells (1,000/well) in 96-well plates were pulse-labeled with 10 µM EdU (37°C, 2 h), followed by DNA synthesis quantification via EdU assay. Following fixation and permeabilization, EdU incorporation was visualized with Alexa Fluor 488 dye from the BeyoClick™ EdU Cell Proliferation Kit (Cat#0071S, Beyotime). Nuclear staining was performed using Hoechst 33342 to distinguish cell nuclei. The results were captured using fluorescence microscopy, allowing for the quantification of EdU incorporation and assessment of DNA synthesis levels in HCC cells.

### Wound-healing assay

In wound healing assays, near-confluent HCC cells (~95%) in 6-well plates were scratched with a sterile pipette tip, PBS-washed to remove debris, and cultured serum-free for ≥24 h. Microscopic monitoring and ImageJ software analysis were used to observe wound closure at designated times (0 and 24 hours).

### Transwell migration and invasion assays

HCC cell motility was quantified through Transwell chamber assays (8-μm pores), assessing both migratory and invasive capacities. Cells (50,000 in 200 μL serum-free medium) were seeded onto Matrigel-coated (invasion) or uncoated (migration) membranes, with 600 μL DMEM/10% FBS as chemoattractant. After 37°C/24 h culture, cells were processed for microscopic evaluation by fixation (4% PFA) and crystal violet staining (0.1%), followed by ImageJ-driven quantification.

### Statistical analysis

Data analysis was carried out utilizing R (version 4.4.1) along with GraphPad Prism 8. Cell proliferation assays, migration, and invasion experiments were analyzed using the ImageJ software. Statistical analyses employed Wilcoxon tests for continuous variables versus chi-square for categorical, log-rank testing for survival, Spearman’s correlation for associations, and ‘rms’ R package (v6.7.0) for nomograms; *in vitro* data expressed as mean ± SD underwent ANOVA with Šidák *post hoc* testing. P ≤ 0.05 was considered statistically significant.

## Results

### Integrative transcriptomic analysis reveals systemic dysregulation of lipid metabolic pathways in hepatocellular carcinoma

**Figure 1:**
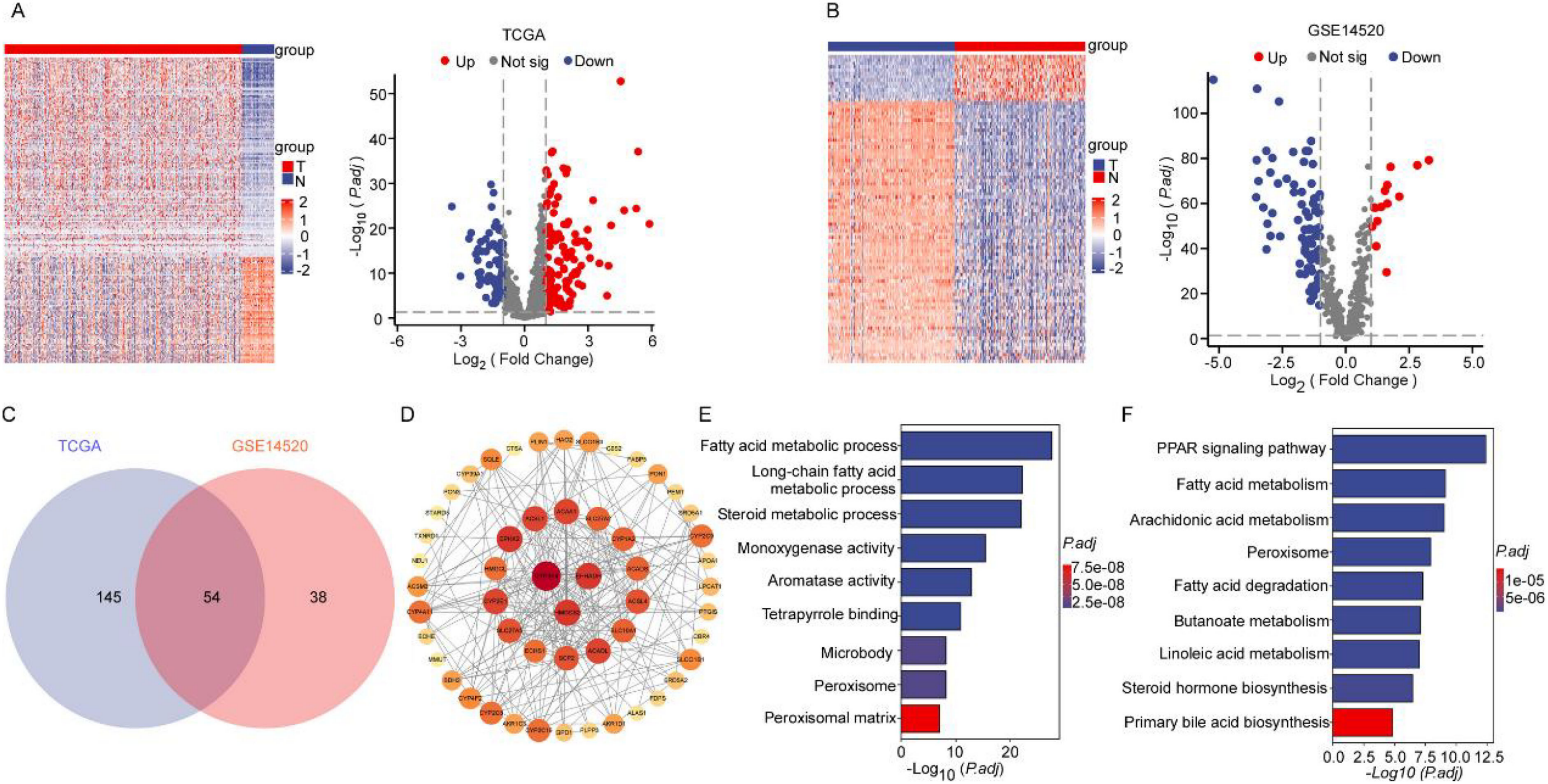
The LMGs and functional analysis. **(A)** DEGs associated with lipid metabolism in LIHC from TCGA. **(B)** DEGs associated with lipid metabolism in LIHC from GSE14520. **(C)** Venn diagram identifying 54 LMGs between the TCGA and GSE14520 databases. **(D)** Interaction network constructed with the nodes with interaction confidence value > 0.95. **(E)** Gene Ontology (GO) enrichment analysis. **(F)** Analysis using the Kyoto Encyclopedia of Genes and Genomes (KEGG). The length of each column represents the count of genes; the shade of color represents the adjusted p value.

Differentially expressed LMGs were identified in two HCC cohorts: TCGA-LIHC (369 tumor and 50 normal samples) and GSE14520 (225 tumor and 220 normal samples). Hierarchical clustering analysis revealed distinct segregation patterns between malignant and non-neoplastic tissues (**Figure 1A**). Volcano plot analysis identified 199 and 92 significantly dysregulated LMGs in the TCGA and GSE14520 datasets, respectively (**Figure 1B**). Intersection analysis using a Venn diagram identified 54 consensus LMGs shared between both cohorts (**Figure 1C**), which were subsequently selected for prognostic model construction. Protein interactome analysis of the 54 candidate LMGs revealed topologically central hubs critically involved in hepatocellular carcinoma pathogenesis (**Figure 1D**). Functional enrichment analysis demonstrated significant pathway associations: Gene Ontology (GO) terms highlighted alterations in lipid homeostasis processes including fatty acid β-oxidation, steroid biosynthesis, and long-chain acyl-CoA metabolism (**Figure 1E**). KEGG analysis linked the LMGs to PPAR signaling, bile acid transport, and arachidonic acid metabolism pathways (**Figure 1F**). This integrative analysis of transcriptomic data demonstrates systematic dysregulation of lipid metabolic pathways in HCC progression.

### Development and validation of a lipid metabolism-based prognostic signature

**Figure 2:**
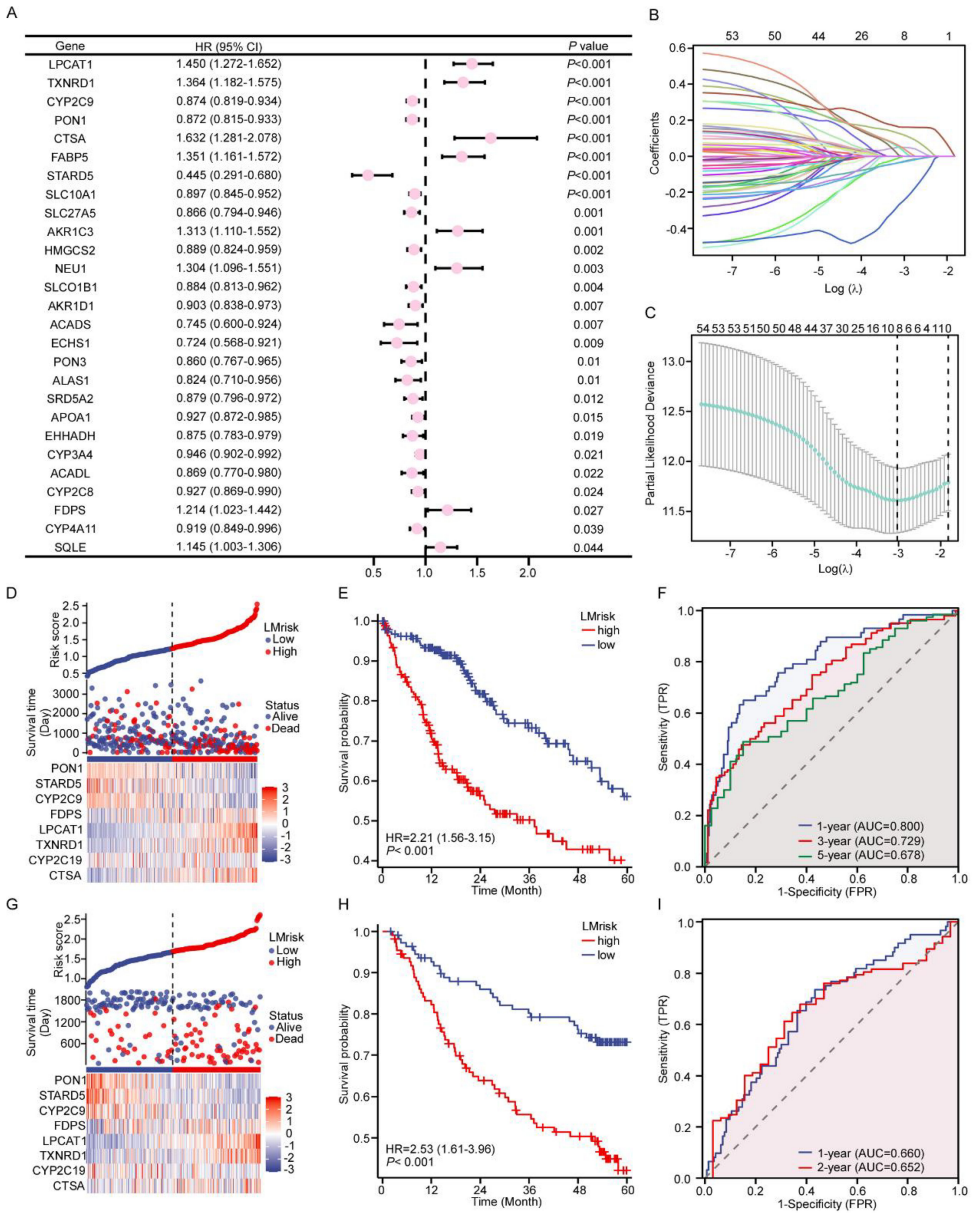
Identification of the LMG-related signature using a machine learning algorithm. **(A)** A total of 27 LMGs were found to have a significant association with the overall survival of HCC patients. **(B)** LASSO Cox regression coefficient analysis was conducted. **(C)** The appropriate lambda value was selected for the LASSO regression model. **(D)** The distributions of the risk score and overall survival status, and the heatmap of the expression of 8 LMGs in the TCGA dataset are shown. **(E)** K-M survival analysis of the LMrisk model in the TCGA. **(F)** Receiver operating characteristic (ROC) curve analysis for the prognostic value of the prognostic model for different years in the TCGA. **(G)** Risk triple plots, including risk dispersion plots, survival time scatter plots, and heatmaps of model gene expression in the GSE14520. **(H)** KM curves of OS in the low- and high-risk subgroups in the GSE14520. **(I)** Receiver operating characteristic (ROC) curve analysis for the prognostic value of the prognostic model for different years in the GSE14520.

Initially, we performed a univariate Cox proportional hazards regression analysis on 54 differentially expressed LMGs using the TCGA-LIHC cohort (n=369 tumors) to develop a reliable prognostic model in HCC. This screening identified 8 risk-associated genes and 19 protective genes with significant survival correlations (**Figure 2A**). Following LASSO regression analysis, the model was streamlined to include eight central LMGs: *PON1*, *STARD5*, *CYP2C9*, *FDPS*, *LPCAT1*, *TXNRD1*, *CYP2C19*, and *CTSA*. (**Figures 2B, C**). The LMrisk was calculated as: LMrisk = (0.051 × *CTSA*) + (0.032 × *CYP2C19*) + (0.121 × *TXNRD1*) + (0.161 × *LPCAT1*) + (0.045 × *FDPS*) - (0.029 × *CYP2C9*) - (0.250 × *STARD5*) - (0.032 × *PON1*). High-LMrisk patients stratified by median cutoff exhibited inferior OS relative to low-risk counterparts. (**Figures 2D, E**). Time-dependent ROC analysis achieved clinically significant predictive accuracy in the TCGA cohort: AUC = 0.800 for 1-year survival, 0.729 for 3-year, and 0.678 for 5-year (**Figure 2F**). External validation using the GSE14520 cohort confirmed the generalizability. Risk stratification maintained prognostic significance with concordant AUCs of 0.660 (1-year) and 0.652 (2-year) (**Figures 2G–I**).

### Clinical translation and predictive modeling of the lipid metabolism signature

**Figure 3:**
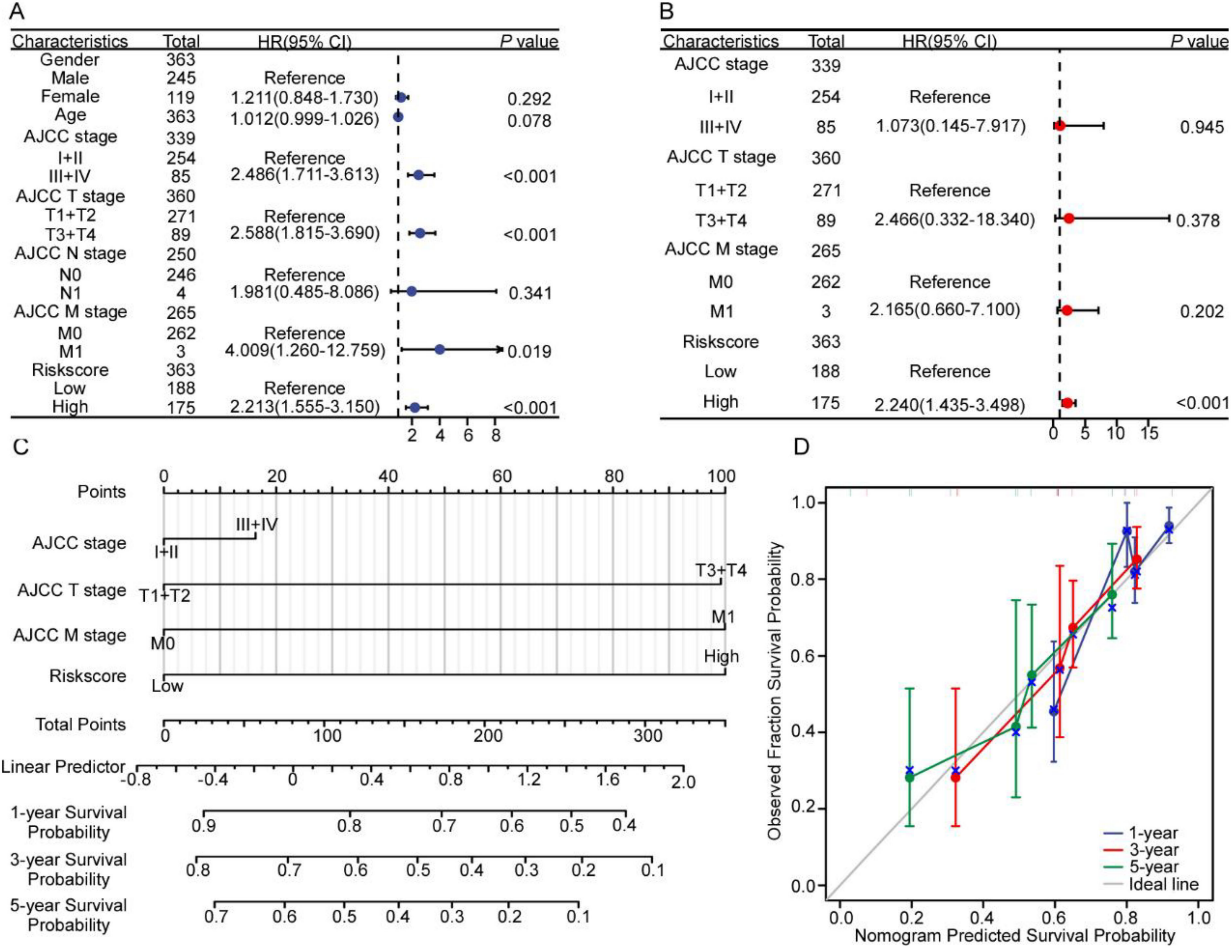
Construction of a nomogram. **(A)** Forest plot of univariate Cox regression for clinicopathological characteristics and the risk score. **(B)** Forest plot displaying multivariable Cox analysis results of variables prescreened by univariate significance (P < 0.05) **(C)** Nomogram to predict 1-, 3- and 5-year survival. **(D)** Calibration curve assessing the nomogram’s predictive accuracy.

Univariate and multivariate Cox analyses incorporating clinicopathological variables validated LMrisk as an independent prognostic predictor for HCC. (**Figures 3A, B**).To enhance clinical applicability, we integrated LMrisk with established prognostic variables to develop a quantitative nomogram for individualized survival prediction (**Figure 3C**). The model demonstrated excellent calibration fidelity, with predicted vs observed 5-year survival probabilities aligning closely (**Figure 3D**).

### LMG signature modulates immune evasion and therapeutic resistance in HCC

**Figure 4:**
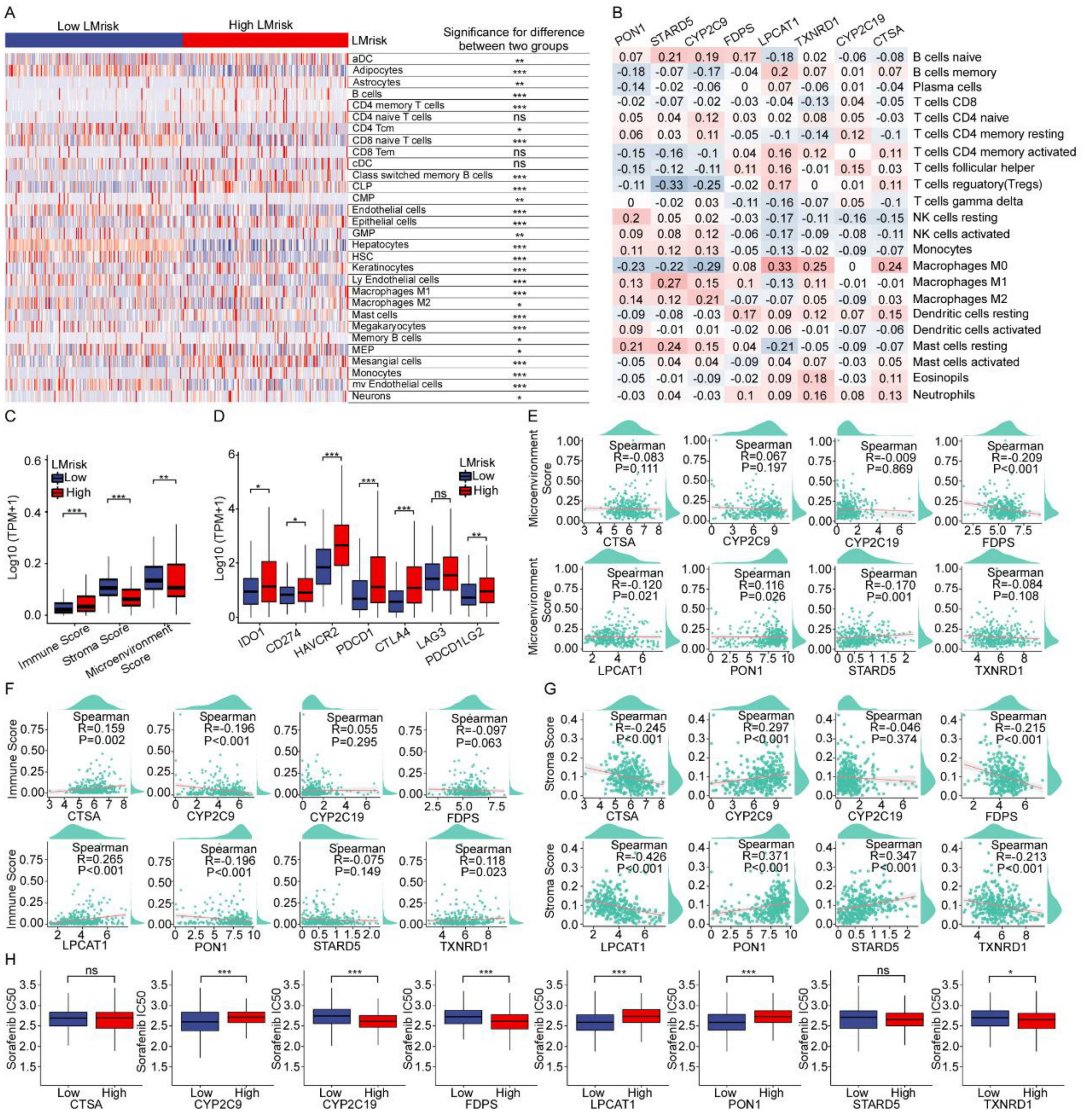
LMGs modulate immune microenvironment and sorafenib resistance in HCC. **(A)** The heatmap visualized normalized infiltration levels of immune and stromal cells. P values were adjusted for multiple comparisons using the Benjamini-Hochberg method. *Adjusted p < 0.05, **adjusted p < 0.01, ***adjusted p < 0.001. **(B)** The correlation heatmap exhibiting the association of the immune cells with the eight characteristic genes. **(C)** Box plot showing scores for immune, stroma and microenvironment. **(D)** Immune checkpoint genes expression levels in the high and low LMrisk groups in TCGA cohort. **(E–G)** The scatter plot showing correlation analysis of the eight LMGs with microenvironment score **(E)**, immune score **(F)** and stroma score **(G)** calculated by xCell algorithm. **(H)** The correlation between sorafenib sensitivity and the expression of eight LMGs. *p < 0.05; **p < 0.01; ***p < 0.001.

Within the pivotal tumor immune microenvironment, xCell algorithm-deciphered immune-stromal infiltration revealed differential abundances of effector memory T cells (Tem) and M1 macrophages between high- and low-LMrisk HCC groups (**Figure 4A**). To further elucidate these findings, we generated a heatmap that visually demonstrated the correlations between our signature genes and the 22 immune cell infiltrations (**Figure 4B**). Subsequently, we evaluated the overall immune score and microenvironment score of HCC patients using the xCell algorithm. A positive correlation was observed between LMrisk and ESTIMATE immune scores in the TCGA cohort (**Figure 4C**). We explored the link between LMrisk and immunotherapy response in HCC to understand the molecular mechanisms affecting altered responsiveness. **Figure 4D** showed that the high LMrisk group exhibited significantly elevated expression levels of *IDO1*, *CD274* (PD-L1), *HAVCR2* (TIM3), *PDCD1* (PD-1), *CTLA4* (CTLA-4), and *PDCD1LG2* (PD-L2) compared to the low LMrisk group. To investigate these findings further, We further assessed Spearman correlations between microenvironment/immune/stromal scores and 8-gene signature expression profiles. These indices exhibited strong correlation patterns according to the results. (**Figures 4E–G**). *LPCAT1* showed a positive relationship with the immune score, while it had a negative relationship with the stroma score. GDSC pharmacogenomic analysis evaluated sorafenib sensitivity—frontline therapy for advanced hepatocellular carcinoma—against eight lipid metabolism genes. Significant positive correlations were identified between mRNA expression of *LPCAT1*/*CYP2C9*/*PON1* and elevated IC50, functionally implicating these LMGs in promoting sorafenib resistance (**Figure 4H**). These findings highlight the possible influence of LMGs on the effectiveness of sorafenib.

### LPCAT1 expression and prognostic value in hepatocellular carcinoma

**Figure 5:**
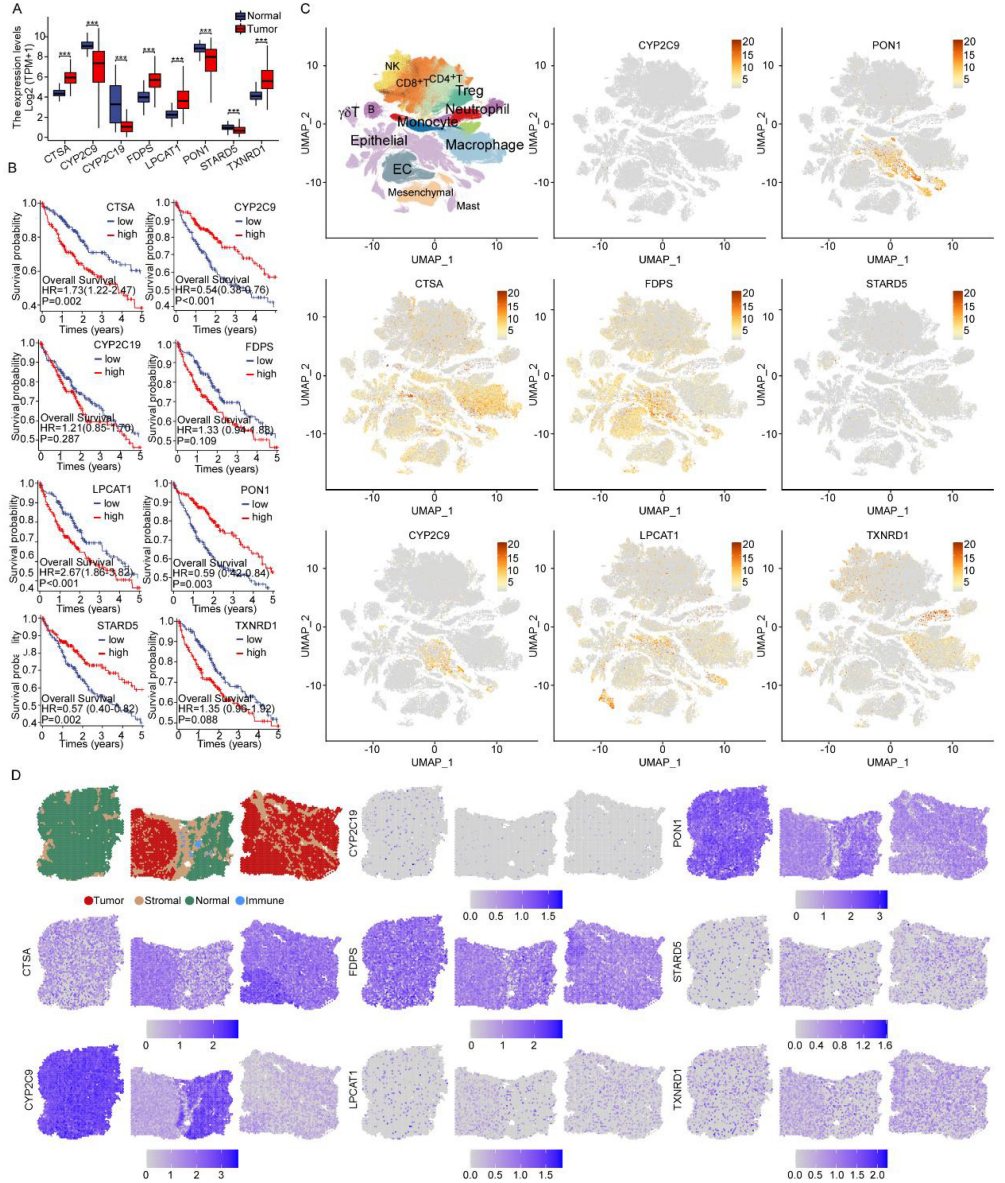
Expression patterns of eight LMGs. **(A)** The 8 LMGs exhibited differential expression between tumor and paired normal tissues in the TCGA-LIHC cohort **(B)** Survival analysis for LIHC patients with different eight LMGs expression. Patients were labeled with high expression or low expression depending on the comparison with the median expression level. **(C)** The cellular localization patterns of eight modeling genes in GSE149614 datasets. **(D)** Spatial analysis of the gene expression of the core LMGs. ***p < 0.001.

Functional assessment of these LMGs involved transcriptional profiling in TCGA-LIHC via bulk RNA-seq. Among them, *CTSA*, *FDPS*, *LPCAT1*, and *TXNRD1* exhibited higher expression in tumor tissues, whereas *CYP2C9*, *CYP2C19*, *PON1*, and *STARD5* showed elevated expression in normal tissues (**Figure 5A**). Survival curves evidenced significantly longer OS in patients with low versus high *LPCAT1* expression (**Figure 5B**). To explore cell-specific expression, single-cell RNA sequencing revealed that *LPCAT1* is predominantly expressed in epithelial cells (**Figure 5C**). Further spatial transcriptome analysis demonstrated the heterogeneity of these core genes’ spatial distribution. *LPCAT1* expression intensified progressively from normal tissue to tumor tissue, indicating functional involvement in disease progression. (**Figure 5D**).

### The expression of LPCAT1 correlates with both tumor development and the immune microenvironment in HCC

**Figure 6:**
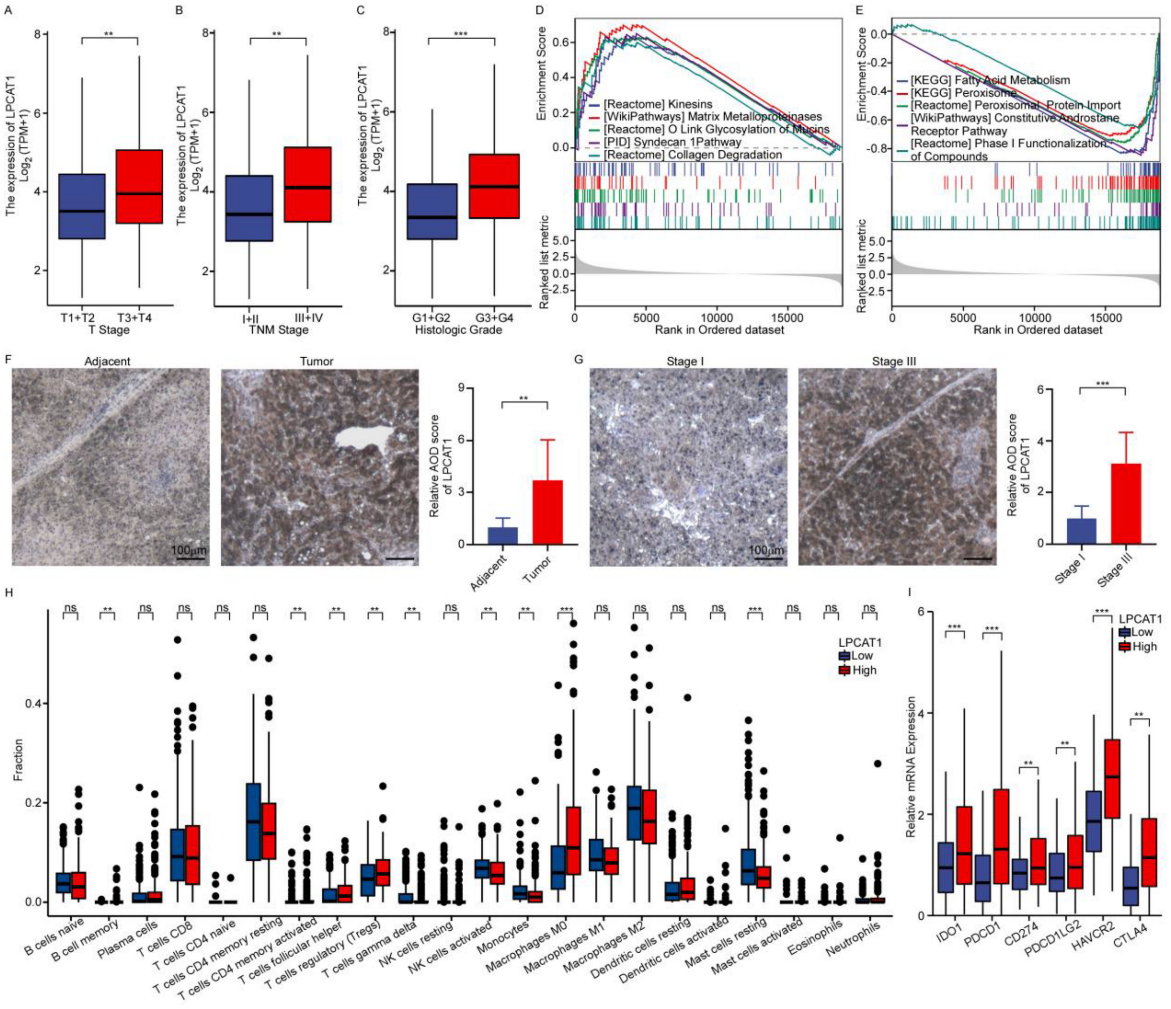
LPCAT1 is upregulated in HCC and shows coordinated associations with ICB activity and immune-stromal infiltration. **(A–C)** Association of LPCAT1 mRNAexpression levels with advanced T stage **(A)**, TNM stage **(B)**, and histologic grade **(C)** in the TCGA-LIHC cohort. **(D, E)** GSEA for samples with highLPCAT1 expression **(D)** and low expression **(E)**. **(F)** IHC shows stronger LPCAT1 protein in tumor tissue than in adjacent non-tumor tissue. Scalebar:100mm. **(G)** LPCAT1 IHC staining was stronger in TNM stage III than in stage I tumors. Scale bar:100mm. **(H)** Box plot showed the ratio differentiation of 21 kinds of immune cells between LIHC tumor samples with low or high LPCAT1 expression relative to the median of LPCAT1 expression level. P values were adjusted for multiple comparisons using the Benjamini-Hochberg method. **Adjusted p < 0.01, ***adjusted p < 0.001. **(I)** Immune checkpoint genes expression levels in the high and low LPCAT1 expression in TCGA. **p < 0.01; ***p < 0.001.

As a key LMG markedly overexpressed in HCC lesions, *LPCAT1* not only predicts poorer OS but also exhibits strong covariation with immune infiltration metrics, suggesting its dual role in metabolic reprogramming and immunomodulation. Based on these compelling findings, we focused our investigation on *LPCAT1*.The expressions of *LPCAT1* were observed to increase progressively with the advancement of T stages, TNM stages, and histologic grades (**Figures 6A–C**). GSEA analysis revealed distinct pathway enrichments between groups with high and low *LPCAT1* expression relative to the median level. The high-expression group showed enrichment in Kinesins, Matrix Metalloproteinases, O link Glycosylation of Mucins, syndecan1 pathway, collagen degradation, while the low-expression group exhibited enrichments in fatty acid metabolism, peroxisome, peroxisomal protein import, constitutive androstane receptor pathway, and phase I functionalization of compounds (**Figures 6D, E**). IHC analysis revealed that LPCAT1 protein expression was significantly higher in HCC tumors compared to matched adjacent non-tumor tissues (**Figure 6F**). Moreover, LPCAT1 expression was significantly elevated in advanced-stage tumors relative to early-stage tumors (**Figure 6G**). Deconvolution of bulk transcriptomes using CIBERSORT quantified tumor-infiltrating immune fractions to assess correlations with *LPCAT1* expression. Our findings indicated that nine types of tumor-infiltrating immune cells (TICs) were significantly linked to *LPCAT1* expression levels (**Figure 6H**). These findings substantiate the hypothesis that *LPCAT1* levels modulate immune activity within the tumor microenvironment (TME), encompassing immune checkpoint regulators and inflammatory mediators. To clarify the molecular mechanisms behind the response to immunotherapy, we examined the link between *LPCAT1* and ICIs in HCC. Tumors with high *LPCAT1* expression exhibited significantly elevated levels of *IDO1*, PD-L1 (*CD274*), PD-1 (*PDCD1*), *CTLA4*, PD-L2 (*PDCD1LG2*), and *HAVCR2* (*TIM-3*) compared to those with low *LPCAT1* expression (**Figure 6I**).

### LPCAT1 overexpression drives proliferative, migratory, and invasive enhancement in HCC

**Figure 7:**
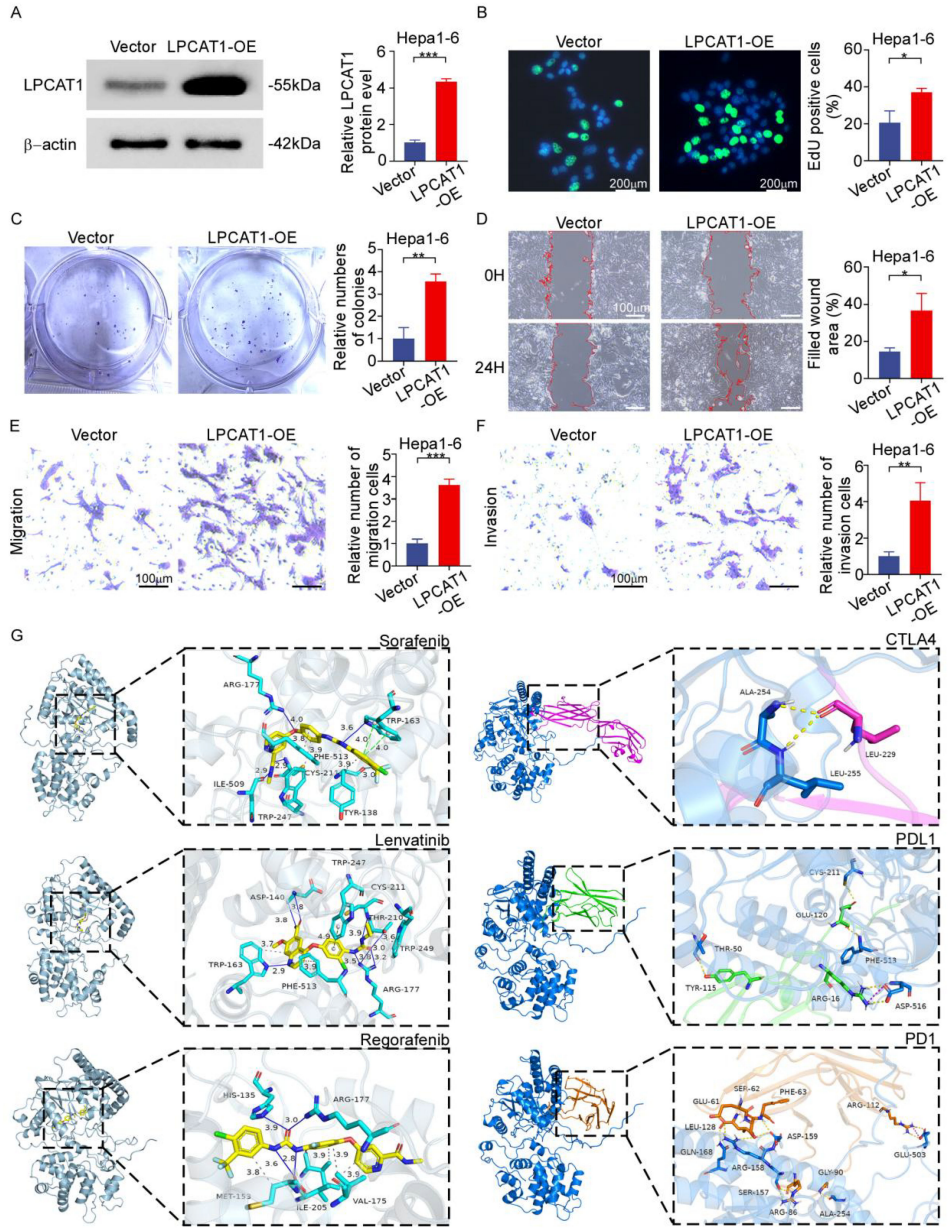
LPCAT1 drives HCC progression and confers therapeutic vulnerability via high-affinity interactions. **(A)** Western blot validation of LPCAT1 over-expression. **(B)** EdU proliferation assay showing increased DNA replication in LPCAT1-overexpressing cells. Scale bar:200μm **(C)** Colony formation assay demonstrating enhanced clonogenic capacity post-LPCAT1 over-expression. Scale bar: 200μm. **(D)** Wound healing assay was performed to evaluate the cell migration of LPCAT1-overexpressed Hepa1–6 cells. Scale bar: 100μm. **(E, F)** Transwell assays with/without matrigel coating for migration **(E)** and invasion **(F)** quantification. Scale bar:100μm. **(G)** The docking conformation and interaction force analysis of LPCAT1. *p < 0.05; **p < 0.01; ***p < 0.001.

*LPCAT1* was overexpressed in a gain-of-function assay to assess its contribution to HCC progression. Western blotting confirmed the elevated protein level of LPCAT1 in Hepa1–6 cells, indicating successful overexpression (**Figure 7A**). The EdU and colony formation assays demonstrated that *LPCAT1* overexpression significantly enhanced the proliferation of Hepa1–6 cells. (**Figures 7B, C**). Functional validation via integrated scratch wound-healing and Transwell assays established *LPCAT1* overexpression as a potent enhancer of Hepa1–6 cell motility and invasion (**Figures 7D–F**). To further investigate the role of *LPCAT1* in human HCC cells, its endogenous mRNA expression levels were first assessed in five human HCC cell lines by qPCR. *LPCAT1* mRNA expression was lowest in Hep3B cells and highest in HepG2 cells (**Supplementary Figure S1A**). Next, Hep3B and HepG2 cells were selected for functional gain- and loss-of-experiments, respectively. Specifically, *LPCAT1* was overexpressed followed by knockdown in Hep3B cells, whereas it was knocked down followed by re-expression in HepG2 cells. The efficiency of transfection was confirmed at both the mRNA level by qPCR (**Supplementary Figures S1B, C**) and the protein level by Western blotting (**Supplementary Figures S1D, E**). Colony formation and EdU assays demonstrated that overexpression of *LPCAT1* in Hep3B cells promoted proliferation, which was reversed by subsequent knockdown (**Supplementary Figures S2A, C**). Conversely, knockdown of *LPCAT1* in HepG2 cells inhibited proliferation, and this effect was rescued by subsequent re-expression (**Supplementary Figures S2B, D**). Wound healing assays showed that *LPCAT1* overexpression enhanced the migration of Hep3B cells, and this promotive effect was attenuated by subsequent knockdown (**Supplementary Figure S2E**). In contrast, *LPCAT1* knockdown impaired the migration of HepG2 cells, which was restored by subsequent re-expression (**Supplementary Figure S2F**). Transwell assays further indicated that *LPCAT1* overexpression in Hep3B cells increased both migration and invasion capacities, and these increases were reversed by subsequent knockdown (**Supplementary Figure S2G**). Accordingly, *LPCAT1* knockdown in HepG2 cells suppressed migration and invasion, and these suppressed phenotypes were rescued by subsequent re-expression (**Supplementary Figure S2H**).

### Molecular docking profiling of LPCAT1 interactions

Targeted and immunotherapy is a first-line treatment for unresectable HCC (31). To computationally explore the potential of LPCAT1 as a therapeutic target, we performed molecular docking analyses with both small-molecule inhibitors and immune checkpoint proteins. Small-molecule docking predicted that three frontline tyrosine kinase inhibitors (TKIs)—sorafenib, lenvatinib, and regorafenib—could form stable complexes within the putative binding site of LPCAT1, with favorable Glide docking scores of -9.436, -7.646, and -8.344, respectively. Protein-protein interaction analysis involved modeling complexes between LPCAT1 and key immune checkpoint proteins (PD-1, PD-L1, CTLA-4). To estimate the binding affinity of these complexes, we employed the MM-GBSA method, which yielded calculated binding free energies (ΔG) of -58.23 kcal/mol for LPCAT1/PD-1, -95.8 kcal/mol for LPCAT1/PD-L1, and -26.86 kcal/mol for LPCAT1/CTLA-4. The detailed interaction interfaces for both the TKI and immune checkpoint complexes are visualized in **Figure 7G**. Collectively, these in silico analyses suggest that LPCAT1 possesses structural features compatible with binding both clinically relevant TKIs and immune checkpoint proteins, positioning it as a potential multi-faceted node within HCC therapeutic networks.

### Molecular dynamics decodes LPCAT1’s role in guiding HCC targeted therapy and immunotherapy

**Figure 8:**
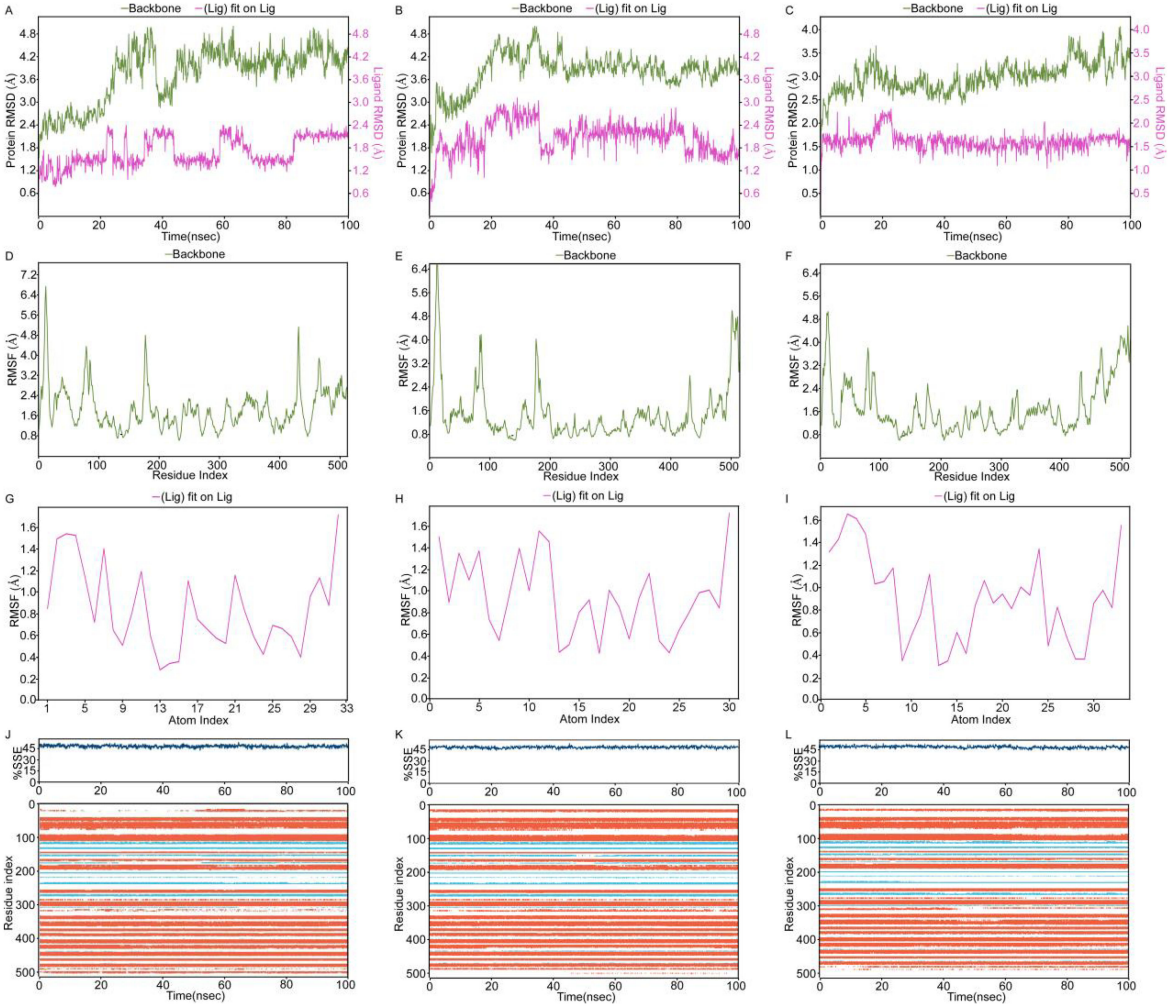
Structural dynamics of LPCAT1-kinase inhibitor complexes. **(A-C)** RMSD plots for **(A)** sorafenib, **(B)** lenvatinib, and **(C)** regorafenib complexes. **(D-F)** RMSF of LPCAT1 in complex with **(D)** sorafenib, **(E)** lenvatinib, and **(F)** regorafenib. **(G-I)** Ligand RMSF for **(G)** sorafenib, **(H)** lenvatinib, and **(I)** regorafenib. **(J-L)** Secondary structure evolution of LPCAT1 bound to **(J)** sorafenib, **(K)** lenvatinib, and **(L)** regorafenib.

**Figure 9:**
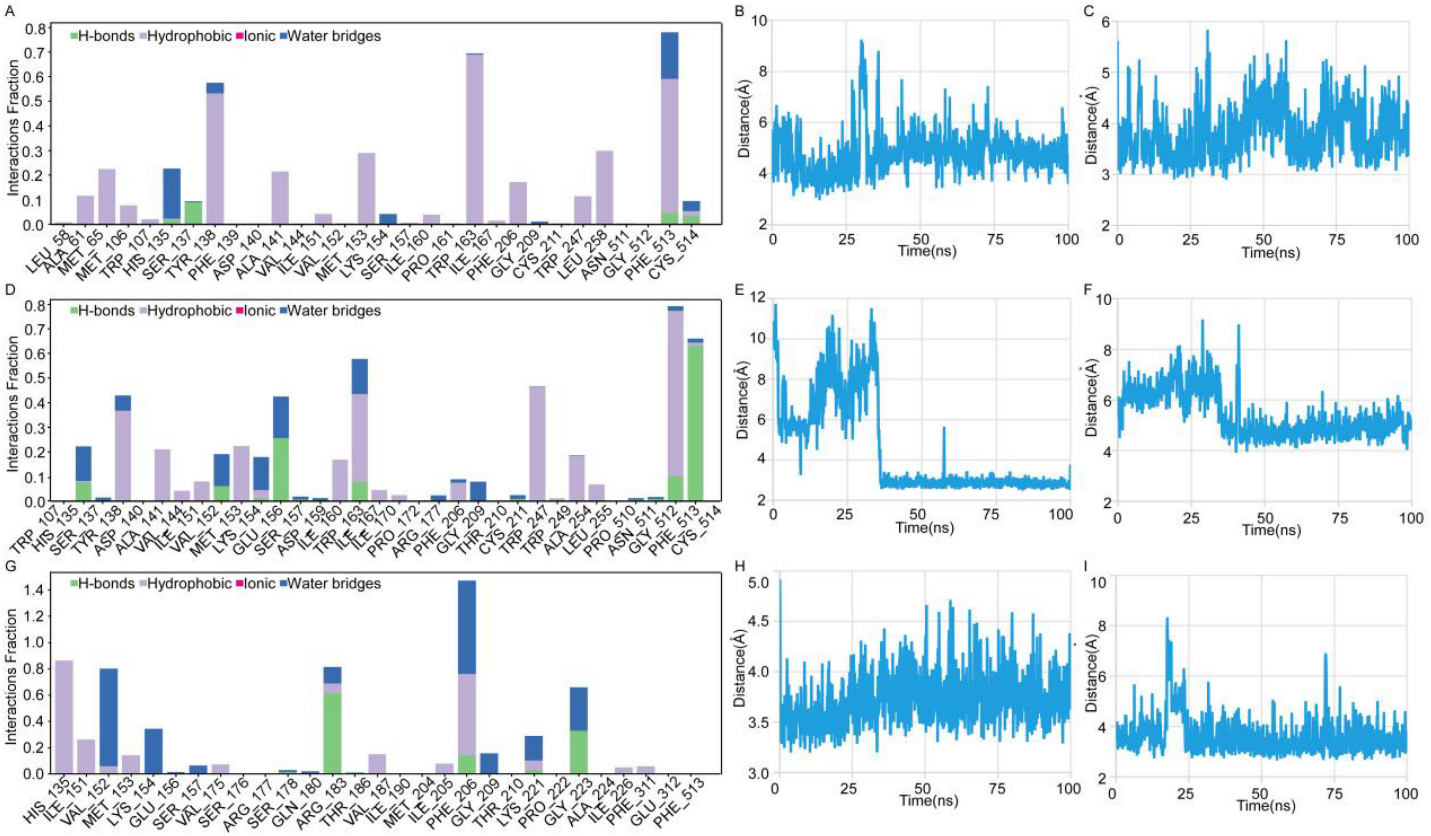
Interaction landscapes and binding metrics. **(A–C)** Sorafenib binding mode: **(A)** Interaction types between sorafenib and LPCAT1. **(B)** TRP163 distance. **(C)** PHE513 distance. **(D–F)** Lenvatinib interaction dynamics: **(D)** Interaction types between lenvatinib and LPCAT1. **(E)** CYS514 distance. **(F)** TRP247 distance. **(G–I)** regorafenib interaction dynamics: **(G)** Interaction types between regorafenib and LPCAT1. **(H)** PHE206 distance. **(I)** ARG183 distance.

**Figure 10:**
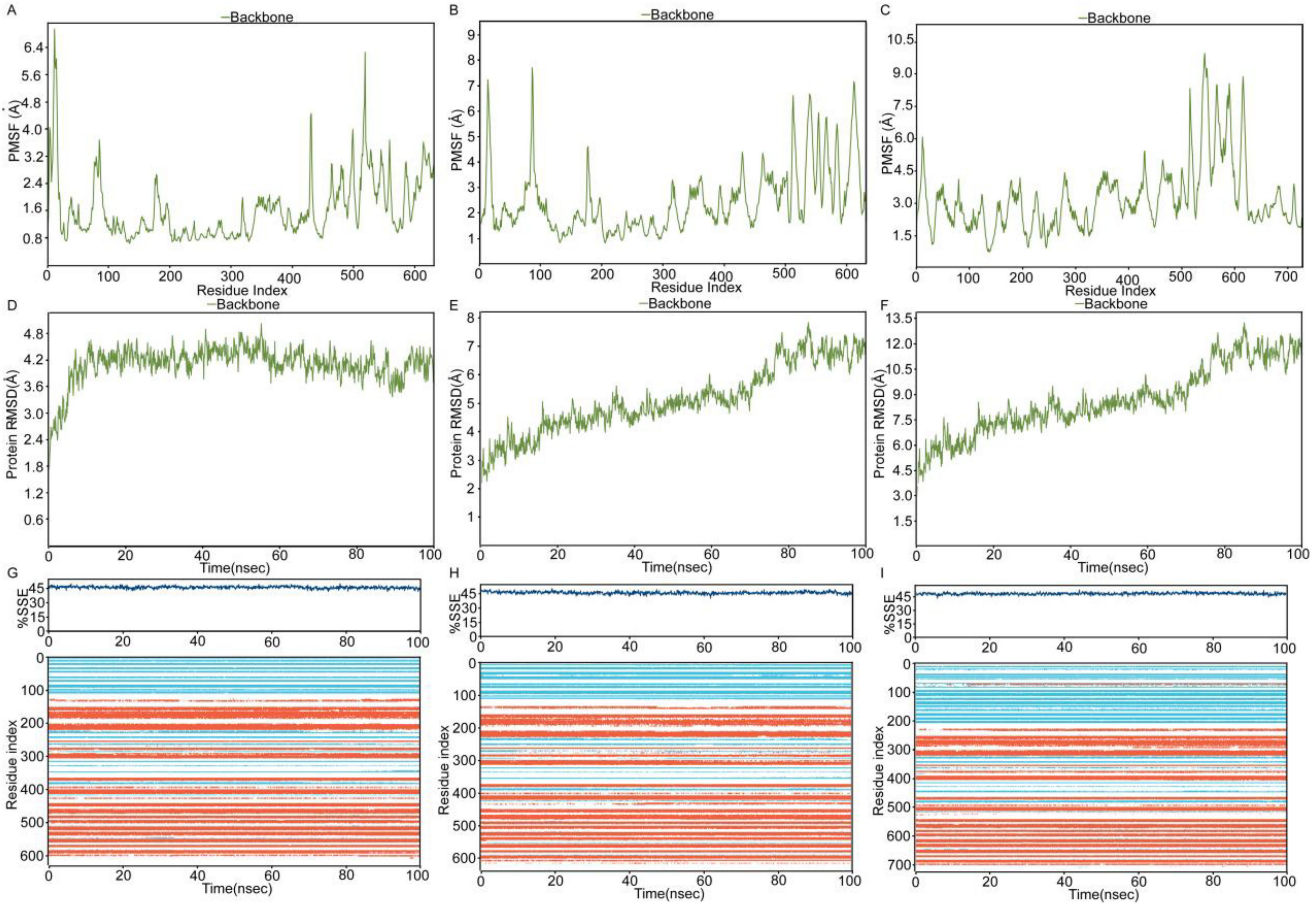
Interaction landscapes and binding metrics. **(A–C)** RMSD plots for **(A)** PD1-LPCAT1, **(B)** PDL1-LPCAT1, and **(C)** CTLA4-LPCAT1. **(D–F)** Interface residue RMSF for **(D)** PD1-LPCAT1, **(E)** PDL1-LPCAT1, and **(F)** CTLA4-LPCAT1. **(G–I)** Secondary structure conservation in **(G)** PD1, **(H)** PDL1, and **(I)** CTLA4 complexes.

Molecular dynamics simulations of LPCAT1 complexes with sorafenib, lenvatinib, and regorafenib revealed small fluctuations in both protein backbone and ligand RMSD values (**Figures 8A–C**). Larger RMSD values indicate greater deviation from the reference structure and lower system stability. In RMSD plots (x-axis: simulation time; y-axis: RMSD), protein and ligand RMSD fluctuations were minor, with ligand RMSD stabilizing at approximately 2-3 Å, indicating relatively stable binding in all protein-ligand systems, particularly for regorafenib. Root Mean Square Fluctuation (RMSF) measures atomic positional deviations from average positions, reflecting regional flexibility (x-axis: residue number; y-axis: RMSF). Lower RMSF values denote reduced fluctuations and enhanced stability. RMSF analysis (**Figures 8D–I**) showed minimal LPCAT1 residue fluctuations (< 4.0 Å) except at flexible terminal regions. Ligand RMSF remained below 2 Å for sorafenib (**Figure 8G**), lenvatinib (**Figure 8H**), and regorafenib (**Figure 8I**), confirming highly stable binding. Secondary structure analysis (**Figures 8J–L**, top: secondary structure content over time; bottom: residue-specific changes; blue: β-strand, orange: α-helix) demonstrated stable conformations without significant alterations during simulations. Interaction analysis identified key residues(**Figure 9A**): sorafenib maintained stable hydrophobic contacts with TRP163 (**Figure 9B**) and PHE513 (**Figure 9C**) at consistently short distances; lenvatinib formed a hydrogen bond with CYS514 and hydrophobic interaction with TRP247 (**Figure 9D**), with distances showing initial fluctuations but stabilizing after ~35 ns (**Figures 9E, F**); regorafenib exhibited persistent hydrogen bonds with PHE206/ARG183, water-mediated hydrogen bonds with GLY223/VAL152, and hydrophobic contact with HIS135 (**Figure 9G**), with stable distances to PHE206/ARG183 (**Figures 9H, I**). MM-GBSA calculations on the final equilibrated frame yielded high binding affinities: sorafenib (-75.7416 kcal/mol), lenvatinib (-68.1357 kcal/mol), regorafenib (-75.286 kcal/mol), confirming strong binding at equilibrium, with sorafenib showing the highest affinity. Molecular dynamics analysis of protein-protein complexes revealed distinct stability profiles: RMSD trajectories for PD1-LPCAT1 (**Figure 10A**), PDL1-LPCAT1 (**Figure 10B**), and CTLA4-LPCAT1 (**Figure 10C**) indicated substantial fluctuations in CTLA4 (suggesting potential instability), while PD1 and PDL1 complexes exhibited comparative stability with PD1 demonstrating superior RMSD consistency (~4.2 Å). Corresponding RMSF plots (PD1: **Figure 10D**, PDL1: **Figure 10E**, CTLA4: **Figure 10F**) further confirmed CTLA4’s elevated residue fluctuations at binding interfaces, contrasting with stable PD1/PDL1 complexes where PD1 maintained the lowest RMSF (~4.0 Å). Secondary structure analysis throughout simulations (PD1: **Figure 10G**, PDL1: **Figure 10H**, CTLA4: **Figure 10I**) showed conserved conformations across all systems without significant structural alterations.

## Discussion

HCC, a highly aggressive and treatment-resistant cancer, poses significant therapeutic challenges due to limited options and unfavorable prognosis (4). In recent decades, significant research has been dedicated to elucidating the molecular mechanisms and prognostic factors of HCC (32, 33). The reprogramming of lipid metabolism exerts multifaceted effects on oncogenesis, including fueling tumor proliferation, accelerating metastatic progression, conferring therapy resistance, dynamically remodeling the immunosuppressive TME, and subverting anti-tumor immunity, thereby positioning it as a critical therapeutic frontier (33, 34). In this study, we established LMrisk for HCC with potential clinical utility in predicting survival outcomes, tumor immune infiltration, and therapeutic responses.

The LMrisk model, constructed from multi-cohort transcriptomic data, proved to be an independent prognostic indicator for HCC. Its eight component genes—*CTSA*, *CYP2C9*, *CYP2C19*, *FDPS*, *LPCAT1*, *PON1*, *STARD5*, and *TXNRD1*—are known to participate in diverse oncogenic processes, including membrane remodeling, drug metabolism, and redox homeostasis (35–43). Incorporating these genes into a unified risk score captures the collective impact of lipid metabolic dysregulation on HCC outcomes. The model consistently demonstrated robust predictive performance, with time-dependent ROC curves yielding AUCs above 0.65 across follow-up periods, further affirming its reliability as a prognostic tool in HCC.

The tumor immune microenvironment promotes HCC progression, in part through lipid metabolic reprogramming that polarizes macrophages toward an M2 phenotype (44–48). Our findings indicate reduced M2 macrophage infiltration in the high-risk group, suggesting diminished antitumor efficacy presumably due to impaired immune cell functionality. Cancer cells evolve diverse mechanisms to evade immune detection and suppress anti-tumor immune responses during progression (45, 46, 48). A key mechanism underlying this immune evasion involves the activation of immune checkpoint pathways (49–54). Our study revealed elevated immune checkpoint expression in high-risk cohorts. Consequently, high-risk patients may exhibit reduced anti-tumor efficacy due to increased expression of immune checkpoints. In conclusion, aberrant expression of LMGs may enable immune evasion, making targeted modulation of metabolic pathways a potential therapeutic strategy for treating HCC.

Immune checkpoint inhibitors combined with targeted therapies represent first-line treatment for advanced HCC (55). The tumor immune microenvironment plays a key role in modulating therapeutic response (45, 55). Integrative bioinformatics analyses in this study identified high LPCAT1 expression as being linked to increased immune scores, enhanced transcription of immune checkpoint markers, and elevated predicted IC50 values for sorafenib. These computational observations suggest a potential association between LPCAT1 and an immunosuppressive phenotype. To functionally explore its role, we performed gain- and loss-of-function experiments in HCC cell lines, which confirmed that modulating *LPCAT1* expression influences proliferative and invasive capacities. Furthermore, molecular docking and dynamics simulations indicated structural potential for LPCAT1 to interact with both tyrosine kinase inhibitors and immune checkpoint proteins, providing a computational basis for its hypothesized dual role in drug response and immune regulation. Collectively, these multilayered analyses position *LPCAT1* not only as a prognostic biomarker but also as a candidate regulator linking lipid metabolism to immune evasion and treatment sensitivity in HCC. Future studies should functionally validate whether targeting *LPCAT1* can indeed overcome resistance and synergize with existing immunotherapies.

Several limitations of our study should be acknowledged. First, the prognostic LMrisk model was developed and validated using retrospective datasets (TCGA and GSE14520). Although it demonstrated consistent performance, prospective validation in independent cohorts—particularly those incorporating modern immunotherapy regimens—is essential to confirm its clinical utility. Second, our immunogenomic and drug sensitivity analyses remain correlative and computational in nature. While molecular docking and dynamics simulations suggest structural interactions between LPCAT1 and immune checkpoint proteins or tyrosine kinase inhibitors, these predictions require functional validation in appropriate experimental systems, such as immune co-culture models or patient-derived organoids. Third, although we have functional validation in HCC cell lines using both gain- and loss-of-function approaches—which confirmed the role of *LPCAT1* in promoting proliferation, migration, and invasion—our study lacks *in vivo* confirmation. Future work employing orthotopic or patient-derived xenograft models would strengthen the translational relevance of *LPCAT1* targeting. Fourth, the association between *LPCAT1* expression and sorafenib resistance is based on in silico pharmacogenomic predictions; experimental validation of chemosensitivity in *LPCAT1*-modulated cells is needed to establish a causal relationship. Finally, while we quantified LPCAT1 protein expression in a limited set of clinical specimens, larger multicenter cohorts with standardized scoring protocols will be necessary to solidify its prognostic and predictive value in HCC patients undergoing immunotherapy or targeted therapy.

In conclusion, our integrated analysis positions *LPCAT1* as a promising lipid metabolism-related biomarker with potential roles in HCC progression, immune modulation, and therapeutic response. Future studies should focus on mechanistic dissection of *LPCAT1* in immune evasion, its functional interplay with checkpoint proteins, and preclinical evaluation of *LPCAT1* inhibition in combination with existing therapies.

## Conclusions

Our study identifies LMGs as potential prognostic biomarkers and establishes a novel risk signature independently associated with overall survival in HCC patients. *LPCAT1* was identified as a key oncogenic driver among eight studied LMG-related genes, accelerating proliferative, invasive, and migratory capacities in HCC. Such effects could attenuate therapeutic responses to targeted therapy and immunotherapy. These findings deepen insights into HCC-associated lipid reprogramming and establish LMGs as innovative prognostic indicators.

## Data Availability

The original contributions presented in the study are included in the article/**Supplementary Material**. Further inquiries can be directed to the corresponding author.
