# Supplementary material for: LPCAT1 as a prognostic biomarker and risk indicator for hepatocellular carcinoma: insights into genes related to lipid metabolism
Source: Front Oncol. 2026 Jan 30;16:1707270. doi: 10.3389/fonc.2026.1707270 (PMC12900719; doi:10.3389/fonc.2026.1707270)
Supplement: Supplementary file 1 [file DataSheet1.docx]

**Table S1.** Effective sequences (5’-3’) of lentivirus plasmids-overexpression.

| Plasmids | | 5’-3’ |
| --- | --- | --- |
| LV- LPCAT1 | atgaggctgcggggccgcgggccgcgggccgccccctcctccagctctggggcaggcgacgcgcggcgcctagcgcccccggggcggaaccccttcgtgcacgagctgcgactgagcgccctgcagaaggcccaggtggctttcatgacgttgacgctgttccccatccggctcctgttcgctgctttcatgatgctgctggcctggccctttgcactcctggcttccctgggacctcctgataaggagccagagcagcccctggccttatggaggaaggtcgtggacttcctgctcaaggccatcatgcgcaccatgtggtttgctggcggcttccaccgtgtagctgtgaaggggcggcaggccctgcctactgaggctgctatcctcaccttggctccacattcctcctactttgatgccatcccggtcaccatgaccatgtcctccattgtgatgaaggcagagagcagagacatcccaatctggggaactctgataagatacatcaggccagtgttcgtgtcccgctctgaccaggactcgcgaaggaagacagtggaggagatcaagcgacgggcacagtcgaatggaaagtggcctcagataatgatttttccagaaggaacttgtacaaataggacctgcctcattaccttcaaacctggtgcgttcattcctggagttcctgtccagcccgtggtgctacgctacccaaacaaattggacaccatcacatggacgtggcaaggacctggagcgttgaaaatcctgtggctcactctgtgccagtttcaaaaccaagtggaaattgaatttctgcctgtgtattgcccttctgaagaggagaagaggaatcctgccctgtatgccagcaatgtgaggcgtgtcatggccaaggccctgggtgtctcagtaaccgactatacatttgaggactgccagctggctctggcagaaggacaacttcgcttgcctgctgacacctgcctgctagagtttgccaggcttgtgaggggcctcggactaaaaccagaaaatcttgaaaaagatctagacaaatattcagaaagtgcgaggatgaagagaggagagaaaatccgccttccagagtttgcggcctacttggaagttcctgtctcagatgcactggaagacatgttctcgctttttgacgagagtggtggtggtgagattgaccttcgtgaatatgtggtcgccttgtctgtggtgtgcaggccatcccagaccttagccaccatccagctggcattcaagatgtacggatctcctgaggatggcagcatagatgaggccaacctgtcctgcatcctcaagactgcactgggtgtatcagaattaacagtgaccgacttgttccaggctattgaccaagaggacaagggaagaatcacctttgatgacttctgcgggtttgcggaaatgtaccccgactacgcagaggactacttgtaccctgatcagacacatttcgacagctgtgcacagacacccccagcaccaactcccaatggcttctgcattgacttcagccctgagaactcagactttgggagaaagaattcttgtaagaaagcggac | |
|  |  | |

**Table S2.** The primers (5’-3’) used in this study.

| Primers for RT-qPCR | | | | 5’-3’ |
| --- | --- | --- | --- | --- |
|  | LPCAT1 | Forward | ACCTGCCTAATTACCTTCAAAC | |
|  |  | Reverse | TCCGCAATACCTATCTTCTCTC | |
|  | β-actin | Forward | GCCGGACTCATCGTACTCC | |
|  |  | Reverse | GTGACGTTGACATCCGTAAAGA | |

**Supplementary Figure 1**


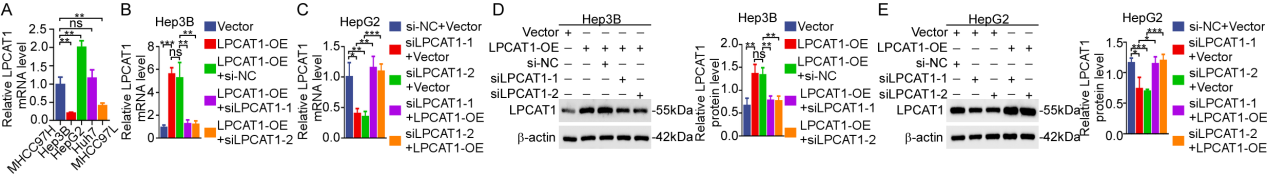


**Supplementary Figure 1**. **LPCAT1 expression profile and modulation validation.** **(A)** qPCR analysis of LPCAT1 mRNA levels in five HCC cell lines. **(B,C)** qPCR analysis of LPCAT1 mRNA levels in Hep3B **(B)** and HepG2 **(C)** cells. **(D,E)** Western blot analysis of LPCAT1 protein levels in Hep3B**(D)** and HepG2**(E)**. *p < 0.05; **p < 0.01; ***p < 0.001.

**Supplementary Figure 2**

**
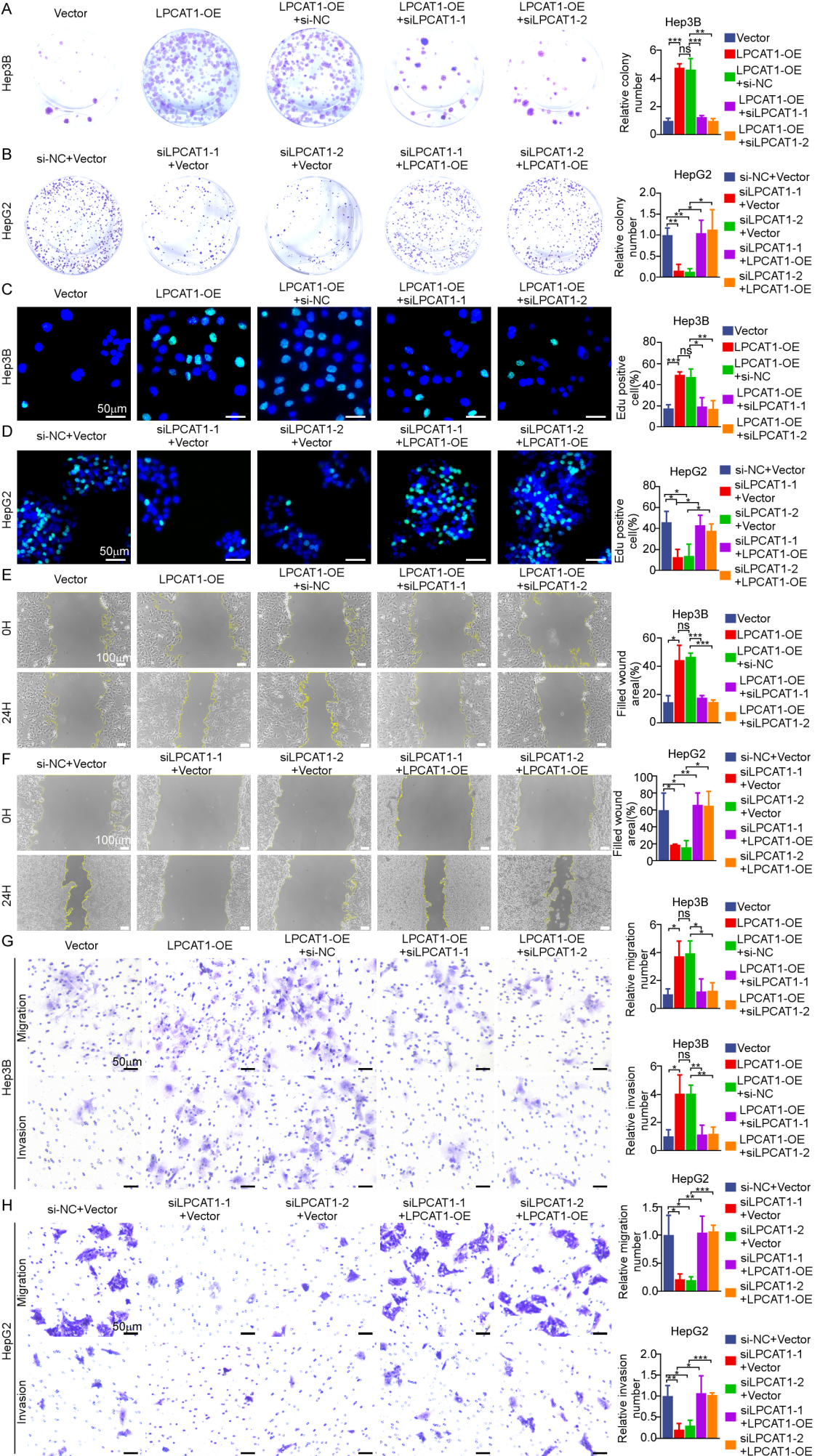
**

**Supplementary Figure 2**. **LPCAT1 regulates proliferation, migration, and invasion in human HCC cells.** **(A,B)** Colony formation assays in Hep3B **(A)** and HepG2 **(B)** cells. **(C, D)** EdU assays in Hep3B **(C)** and HepG2 **(D)** cells. Scale bar:50mm. **(E, F)** Wound healing assays in Hep3B **(E)** and HepG2 **(F)** cells. Scale bar:100mm. **(G, H)** Transwell migration and invasion assays in Hep3B **(G)** and HepG2 **(H)** cells. Scale bar:50mm. *p < 0.05; **p < 0.01; ***p < 0.001.
